# The impact of large-scale afforestation on ecological environment in the Gobi region

**DOI:** 10.1038/s41598-021-93948-5

**Published:** 2021-07-13

**Authors:** Zhengyi Yao, Jianhua Xiao, Xixi Ma

**Affiliations:** 1 Northwest Institute of Eco-Environment and Resources, Chinese Academy of Sciences, Lanzhou, 730000 China; 2 University of Chinese Academy of Sciences, Beijing, 100049 China

**Keywords:** Ecology, Environmental sciences, Natural hazards

## Abstract

In recent year, there has been large-scale afforestation in the gobi region of western Inner Mongolia, China. However, this area has low annual precipitation of 35–55 mm, and a high annual evaporation capacity of 3842 mm. Further, water resources in this region are scarce and cannot support the sustainable growth of shrubs. Thus, without effective irrigation, the shrubs cannot survive and ultimately, large-scale afforestation in the gobi region will destroy “black vegetation”. The surface of this area is covered by dense gravel (65.43–82.08%; average of 77.14%) as a result of long-term erosion caused by strong winds. The sediments underlying the gravel layer are rich in sand (60.34–87.51%) and silt (11.26–35.18%). Once the surface gravel layer is destroyed, the underlying sand and silt expose and increase dust supply, and result in increased intensity and frequency of dust storms. Thus, large-scale afforestation in the gobi region is an ecological disaster for these very dry lands.

## Introduction

**Figure 1:**
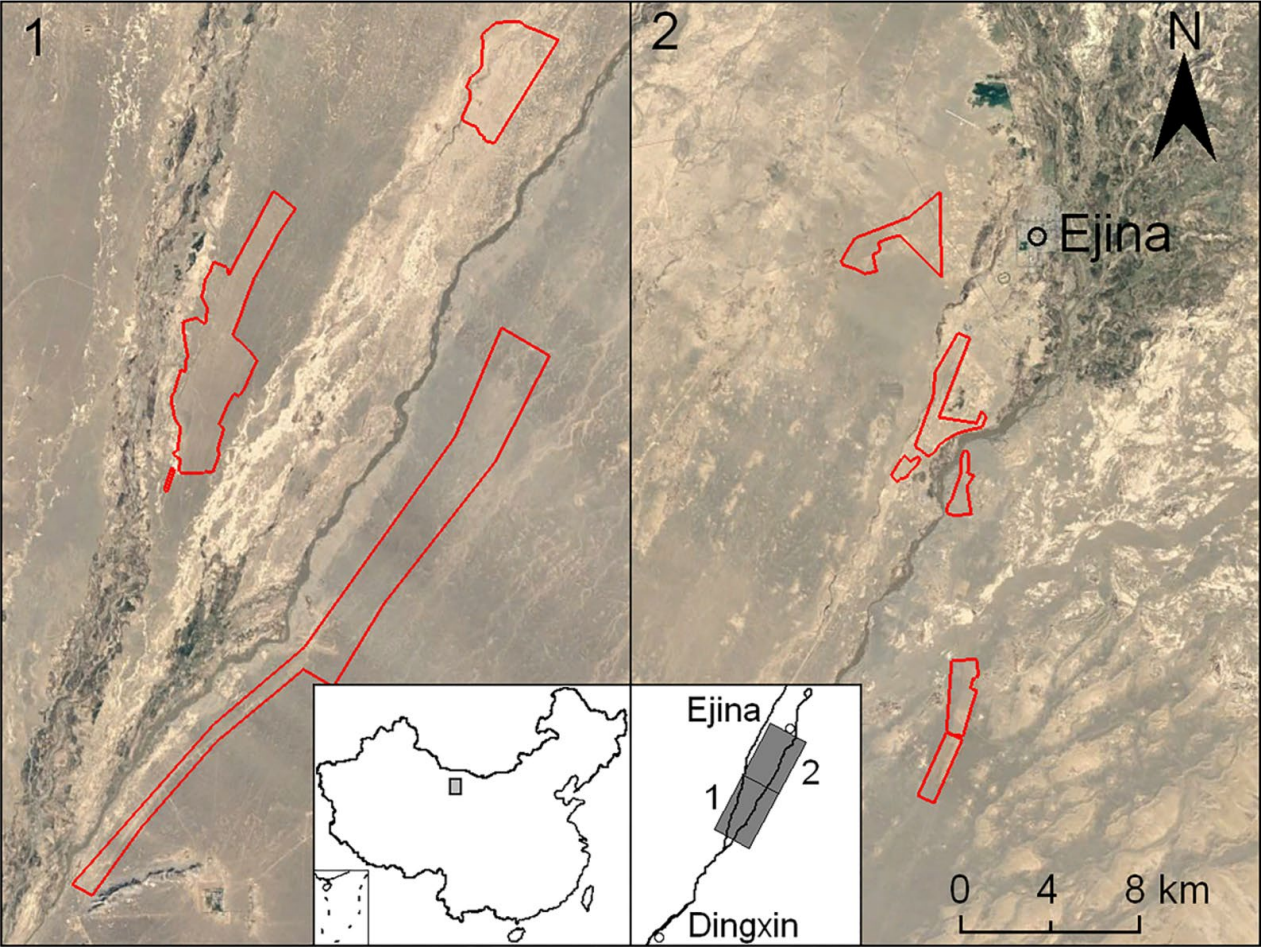
Satellite image of afforested area in the gobi region, Ejina Qi, Inner Mongolia, China. Red-lined areas indicate locations where shrubs (*Haloxylon ammodendron*) were planted. The maps were generated using ArcGIS software version 10.3 (https://www.arcgis.com/index.html) and insert satellite images are downloaded from Google Earth Pro software version 7.1.7.2606 (https://www.google.com/earth/versions).

The gobi is defined as a region where surfaces are composed of coarse angular or rounded particles, with a typical thickness of one or two stone layers and set on or in deposits of sand, silt or clay^1,2^. This is also referred to as “stone pavement”, “desert pavement”, “gibber plain” and “hammada”^3^. The lower reaches of the Heihe River’s alluvial fan in Ejina Qi, western Inner Mongolia, is a typical gobi region in China, with a total area of 3 × 10^4^ km^2^, two-thirds of which is covered by gravel, and one-third by sand and silt. It is one of the main dust sources in East Asia^4^. In recent year, there has been large-scale afforestation in this region. According to an incomplete survey (as estimated from satellite images), between 2013 and 2018, approximately 100 km^2^ of the gobi region was afforested with 5 × 10^6^ shrubs (*Haloxylon ammodendron*) (Fig. 1).

## Water resources cannot support sustainable growth of shrubs

**Figure 2:**
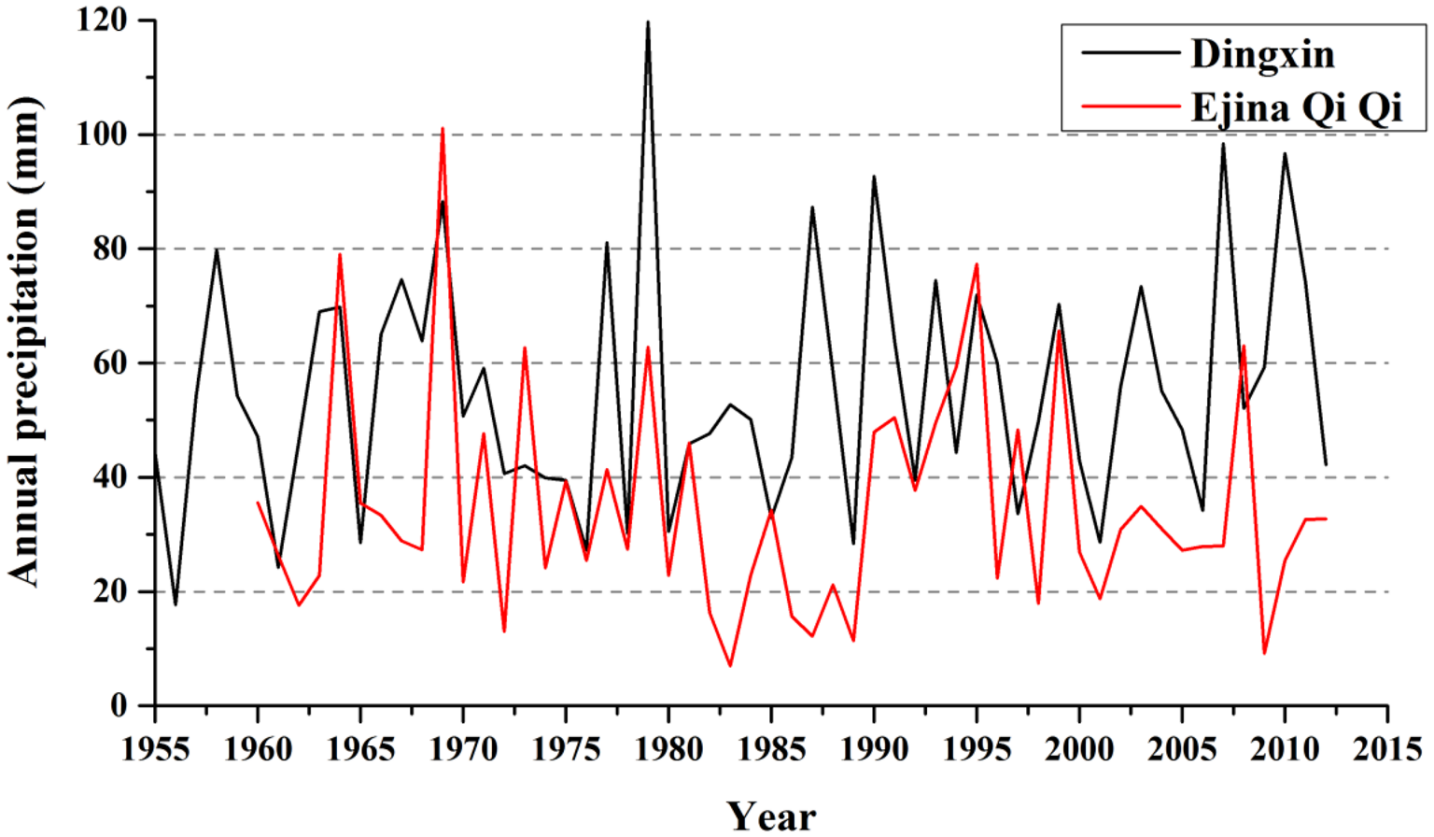
Variations in annual precipitation in Ejina Qi and Dingxin station.

Ejina Qi has a temperate arid climate and encompasses dry and very dry land, which receives annual precipitation of 35–55 mm and has an annual evaporation capacity of 3842 mm. Recent 60-year meteorological records showed that the maximum annual precipitation in Dingxin and Ejina Qi Stations was 119.7 mm (1979) and 101.1 mm (1969), respectively, and the minimum values were 17.7 mm (1956) and 7 mm (1983), respectively. The data also showed seriously low precipitation in several consecutive years, for example, during 1982–1989, Ejina Qi received 7–34.3 mm precipitation, with a mean value of 17.6 mm, and during 2000–2007, it received 18.7–34.9 mm with mean value of 28.2 mm (Fig. 2). This phenomenon is extremely unfavorable to the survival of vegetation.

Moisture provided by precipitation infiltration is the main water source that can be used by vegetation^5^. Annual precipitation in the gobi region is concentrated and short, usually occurring in 4–5 isolated precipitation events. Maximum daily precipitation is about 12 mm. Field investigation showed that a single precipitation event of 24.7 mm wets only 6–7 cm of the sand layer^6^. Intense evaporation removes surface water almost immediately, leaving only a small amount of water to infiltrate to lower soil layers, and little moisture is available in the lower soil layers of this area. Normal precipitation cannot therefore improve the water condition of such sandy land. So only the very sparse drought- resistant can survival here.

*Haloxylon ammodendron* is a drought-resistant shrub species with a well-developed root system, adapted to very dry land. Its main root can reach depths of more than 2 m, with the deepest roots being able to reach groundwater at depths of 4–5 m. This species can survive in conditions with the moisture content of below 2%, but the moisture content below 1% can cause serious recession of the shrubs^7^.

The Heihe River is the only water source in this area and has received only 5.34 × 10^8^ m^3^ water per year since 2002. The lateral supplement of this river to its alluvial fan is limited to several hundred meters on either side of the river channel. The sites where shrubs were planted, however, are 1–6 km away from the Heihe River, with a groundwater level at depths of 5–10 m, which even the longest roots of *H. ammodendron* cannot reach. Therefore, the planted shrubs cannot use this underground water.

**Figure 3:**
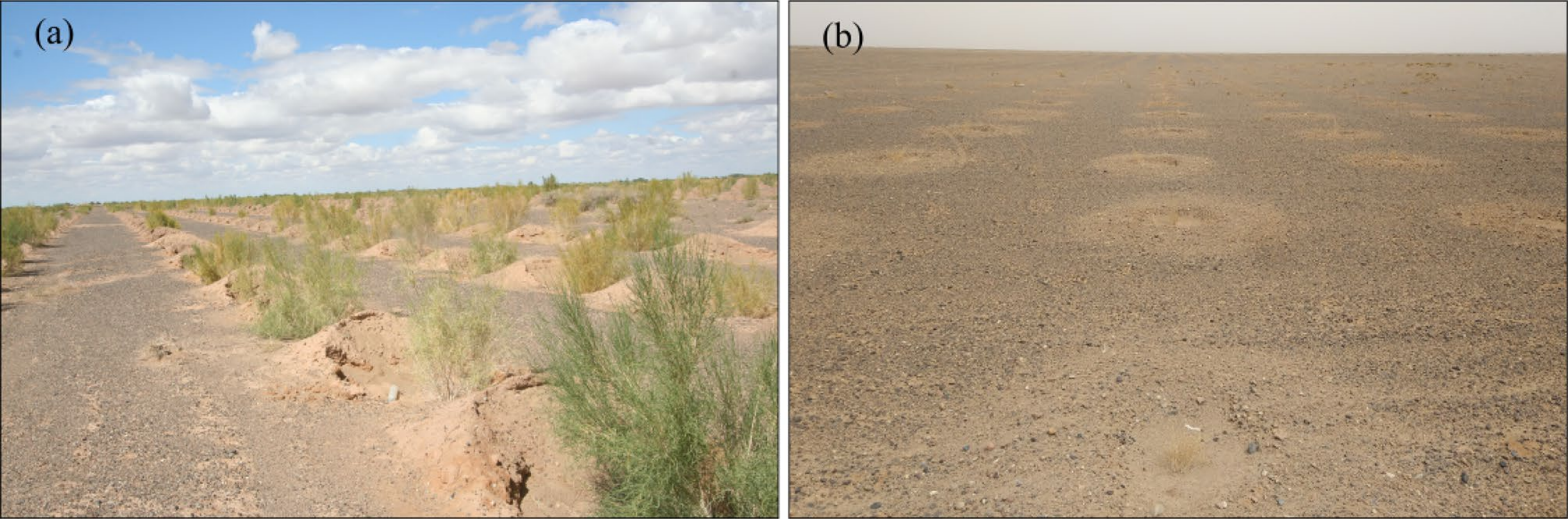
*Haloxylon ammodendron* planted in gobi region in Ejina Qi. Shrubs grew well under irrigated conditions (**a**) but withered and died without irrigation (**b**).

As we estimated from satellite images and field investigation, the density of the newly planted shrubs was 50,000 plant/km^2^. Based on experience, the water consumption of *H. ammodendron* is about 0.3 m^3^/year per plant. This means that each shrub consumes about 15 mm of precipitation per year. Considering this utilization efficiency, the annual precipitation of 35–55 mm cannot support the sustainable growth of this shrub. Therefore, continuous irrigation is key to the shrubs’ survival. With effective irrigation, the shrubs could survive the first two years with good growth conditions (Fig. 3a). However, irrigation was cost- and labor-intensive, and once it stopped, the shrubs withered and died over the next years (Fig. 3b).

## Destroy black vegetation

**Figure 4:**
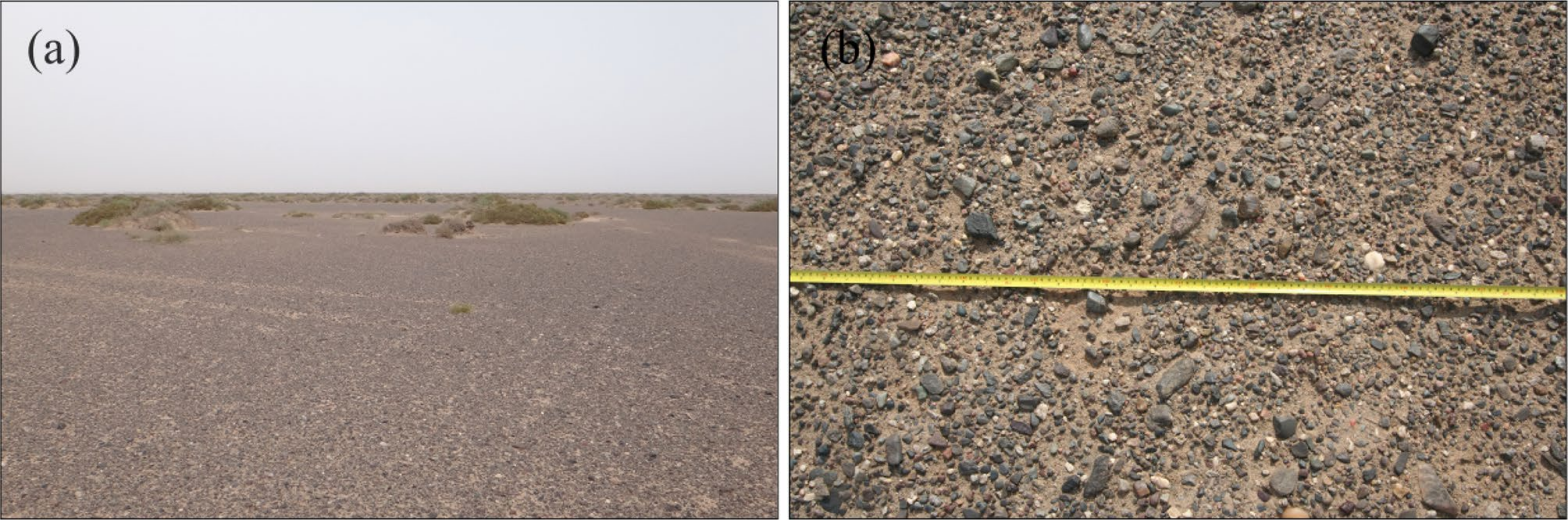
Original surface of gobi region in Ejina Qi, “Black vegetation” covers the surface of the land (**a**) and has grain sizes of 2–40 mm (**b**).

**Figure 5:**
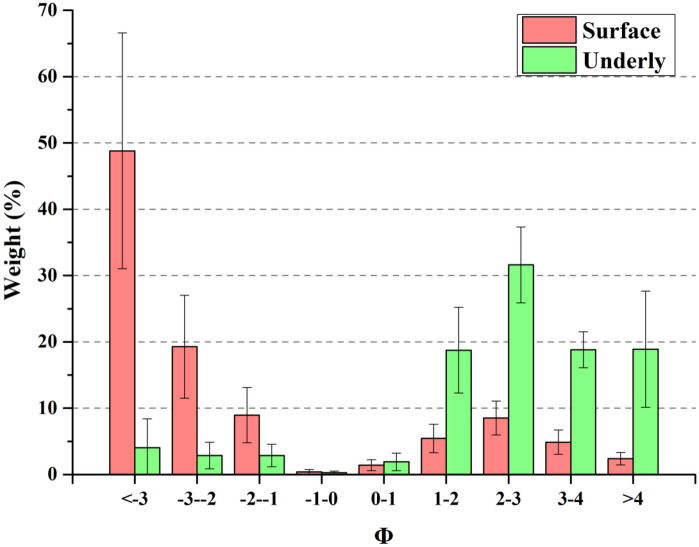
Grain size distribution of sediments on surface and underlying soil layers of gobi region in Ejina Qi.

The most serious environmental consequences of planting shrubs in the gobi region is the destruction of “black vegetation”, increase of dust supply, and increased intensity and frequency of dust storms^8–11^. Eroded by long-term strong winds, the alluvial fan of the Heihe River has changed into desert area, known as the Central Gobi Desert. Gravel is the basic characteristic of this part of the desert (Fig. 4). Erodible fine sand is transported away from the desert, while non-erodible gravel and coarse sand are concentrated on the surface, protecting underlying soil from erosion^12–14^. According to our field investigation, the average gravel grain size is 4.90 mm. Gravel (with a grain size of > 2 mm) covers 65.43–82.08% (with an average of 77.14%) of the study area, followed by sand (16.19–31.08%, with an average of 20.48%), and silt (1.02–3.50%, with an average of 2.39%). Of sediments with a grain size of > 2 mm, about 58.33% comprises medium and coarse pebbles (8–32 mm), 25.03% comprises fine pebbles (4–8 mm), 11.61% comprises very fine pebbles (2–4 mm), and few very (5.03%) coarse pebbles or cobbles (> 32 mm) (Fig. 5). Therefore, the gobi region has a good aerodynamic bed surface for the rapid transport of large amounts of sand^9^. The pores between gravel particles can trap and fix sand. This layer of gravel and coarse sand plays a significant role in the area’s ecology and landform, decreasing the dust emissions known as “black vegetation” to the gobi region.

**Figure 6:**
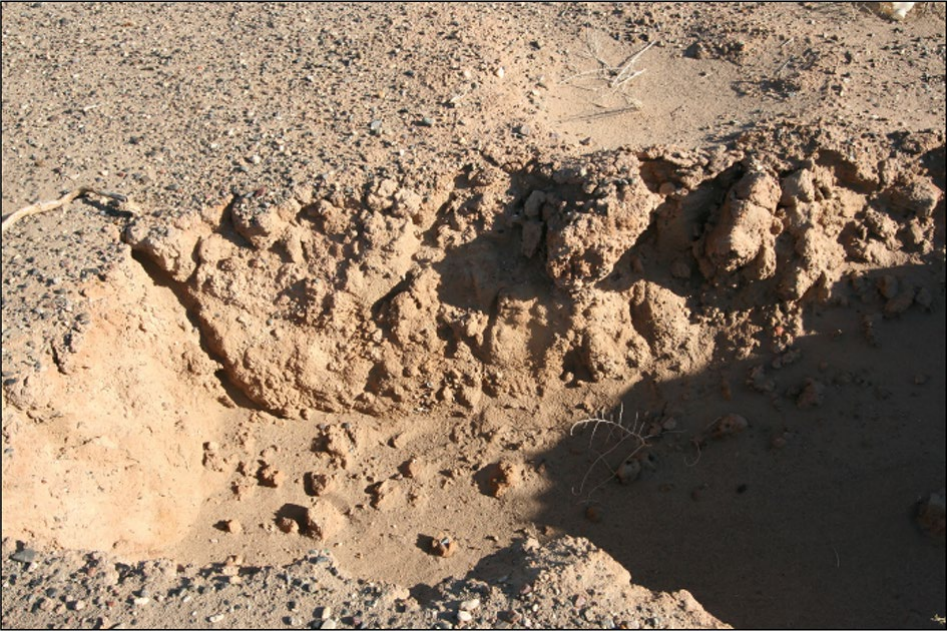
Underlying alluvial sand of gobi region in Ejina Qi.

The underlying alluvial sediments of the study area consist of a mixture of coarse and fine grains (Fig. 6). The sediments underlying the gravel layer mainly comprise sand (60.34–87.51%), silt (11.26–35.18%), and very fine gravel (1.23–16.98%), with mean values of 71.08, 18.89, and 10.03%, respectively. The mechanical composition and sedimentary structure of these materials are not only a reflection of the original sediments in the gobi region but also contains clues about transportation force, migration laws, and sedimentary processes.

**Figure 7:**
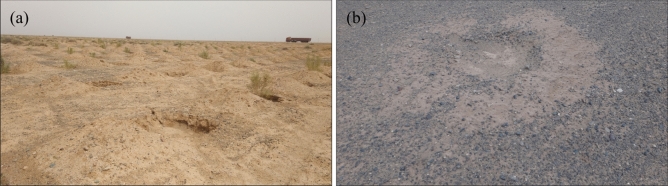
Surface of gobi region in Ejina after pits for shrubs were dug by excavation (**a**) and auger digging (**b**).

According to our field survey, two main excavation methods were used to dig the pits in which the shrubs were planted in the gobi region: auger and excavator. Augers were used to dig pits with diameters and depths of 0.6 and 0.9 m, respectively, and volumes of 0.25 m^3^. The volumes of pits dug via excavators were 1.0–1.4 m^3^ for larger pits and 0.45–0.6 m^3^ for smaller pits. Instead of the pits being backfilled immediately, they were left to fill up with aeolian sand before the shrubs were planted. This method was used to replace the soil (the original soil contained salt and alkali) (Fig. 7). All the soil excavated from the pits was thus left outside, and the sand particles were blown away by the wind, forming sand movement, while the powdered particles formed dust storms.

## Discussion and conclusions

**Figure 8:**
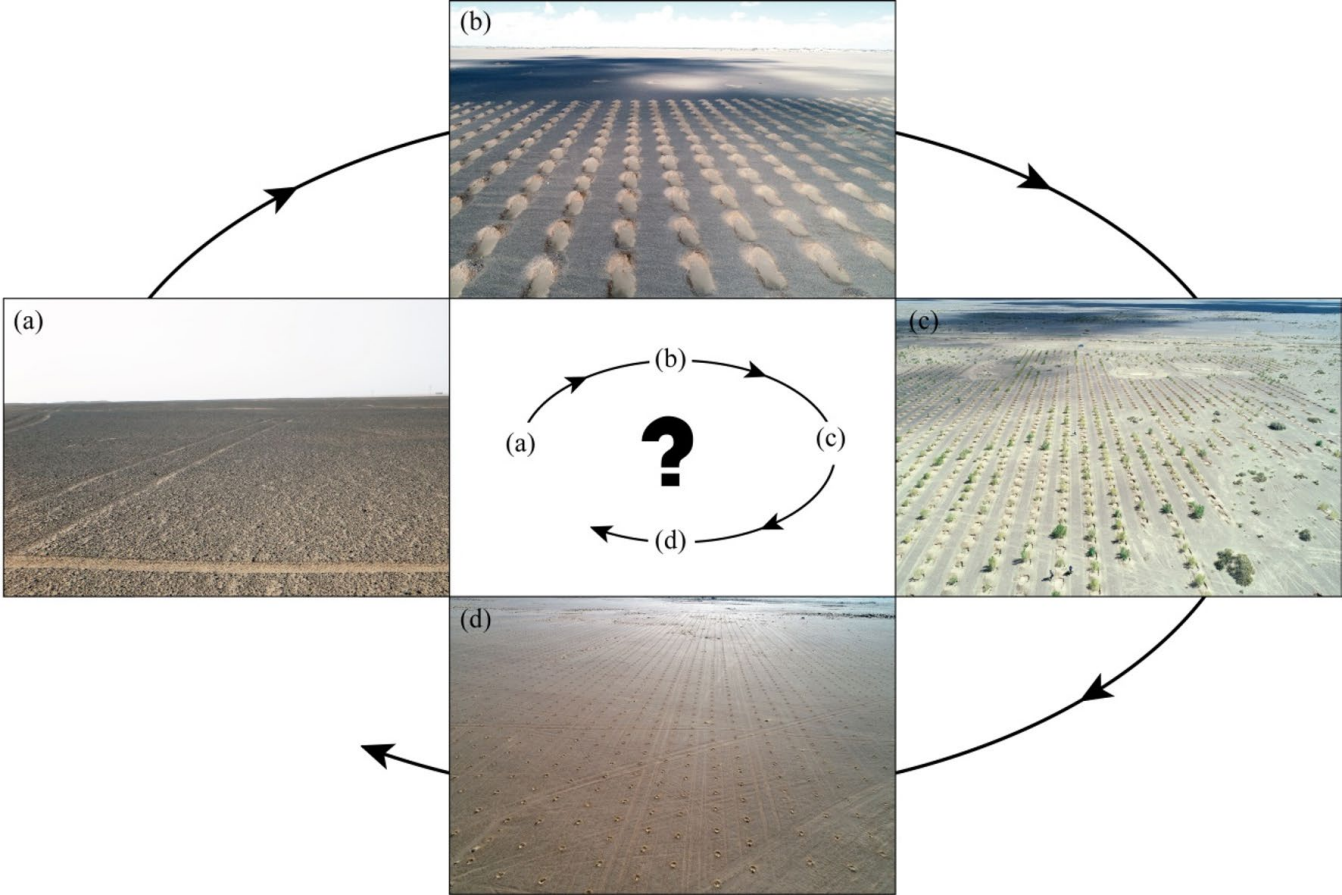
Diagram of different development stages of large-scale afforestation in the gobi region (**a**: original ground surface; **b**: holes dug for afforestation; **c** the living trees planted; **d**: ground surface when the trees are dead).

The gobi region ecosystem has low stability because of its single species composition and simple structure (Fig. 8a). Large-scale shrub planting destroyed the original stable state (Fig. 8b) and resulted in another stable state via self-adjustment. In this process, the planted shrubs deteriorated the original ecosystem by competing for water and a chain reaction may ensue, leading to greater ecological problems. The original intention of the large-scale planting of shrubs was to maintain regional ecological balance, protect biodiversity, and fix sand, thus improving the environment (Fig. 8c). However, given the poor choice of the planting location, the expected results were not achieved. In fact, the opposite results of the original good intentions were achieved (Fig. 8d).

China has a large expanse of arid areas, and has suffered from droughts for a long time. Land afforestation has been at the forefront of China’s policy principles, and there are government departments specializing in this field. In recent years, the Chinese Government has recommended a series of major strategies, for example, the "construction of ecological civilization" and "lucid waters and lush mountains are invaluable assets", and also promoted greening projects, including "Three North Shelterbelt Project", "Beijing-Tianjin Sandstorm Source Control Project", and the "Natural Forest Protection Project". More recently, desert greening has been conducted by people and enterprises, for example, the Ant Forest and Society of Entrepreneurs & Ecology (SEE). As a result of these projects and initiatives, China's greening has contributed to global greening totals^15,16^. For afforestation, China's policy departments have recommended the principles of "sticking to local conditions, suitable land for green, suitable trees for trees, suitable shrub for shrub, suitable grass for grass" and promoting the overall protection of "Mountain-River-Forest-Farmland-Lake-Grass-Desert system", with particular references to desert. Their goal is to scientifically promote afforestation of the land and to clarify "where to afforest, what to afforest, how to afforest, how to manage". However, problems arise very easily when grassroots executors are involved.

The total area of the gobi region in China is approximately 56.95 × 10^4^ km^2^, accounting for 13.36% of the national area, and is primarily distributed in the northwest extreme arid regions^17^. As mentioned above, gobi refers to a special arid landform that has a notably low water supply and is unsuitable for growing trees and shrubs. As an important natural landform, the gobi plays a key role in ecological protection; hence, its reference as "black vegetation". However, there is a lack of understanding of the gobi, and it is often regarded as an area that needs to be greened or reformed. However, gobi, as an extremely arid region, is a fragile ecosystem. Once the gravel on the gobi surface is destroyed, it could lead to a series of ecological and environmental problems. Therefore, afforestation in arid areas is both a scientific and technical issue which must be conducted according to different regional characteristics, rather than by blindly planting trees in unsuitable areas. This study aims to attract more attention from the government forestry department and implementation personnel involved in afforestation activities so as to revise relevant policies. In response to the findings of this study, we have several recommendations: (1) it is necessary to popularize the understanding of scientific greening within the general public; (2) scientific understanding of the gobi needs to be increased, and awareness must be raised to promote its protection; (3) afforestation projects and management must be scientifically and systematically improved to ensure long-term effectiveness, and; (4) restoration and protection measures should be taken immediately in the gobi regions that have been afforested or destroyed.

One of the most important causes of all these problems is the implementation of national policies on subsidies for greening and planting trees in desert areas. According to our survey, personnel who specifically plant trees and engage in afforestation are businessmen, farmers, or others, with most of them being businessmen from abroad, and only a few being local people. All the personnel are more concerned about the subsidies than greening and planting trees itself. According to the policy, they will receive majority of the subsidy if the planted trees live for three years, irrespective of whether the trees survive after that. Therefore, to guarantee the survival of the planted trees for three years, they even use water tankers to carry water to the trees from a great distance. However, after three years, the people stop watering the trees planted in the Gobi region, thereby leading to the death of trees after a few years as they cannot survive only on natural precipitation and groundwater. In pursuit of maximum profits, these businessmen will pursue larger areas for planting trees, which will cause further damage to the ecological environment in the Gobi region. Based on the current situation, we propose the following suggestions: (1) Trees that are planted must be monitored over a long time period, which will greatly reduce the short-term profit motive of the people engaged in planting trees. (2) We must plan greening and planting trees according to local conditions, respecting the laws of nature. Not all areas should be greened; moreover, we should not plant trees, especially in the gobi region, where planting trees can possibly destroy the gobi ecological environment, which is a very fragile desert ecosystem. (4) Personnel responsible for the destruction of the gobi ecological environment by unscientific greening and planting of trees must be obligated to restore the surface conditions of the gobi to prevent the aggravation of wind erosion and desertification, which will increase their awareness of environmental protection and receive punishment for environmental damage.
